# Prophylactic use of levosimendan in preoperative setting for surgical repair of congenital heart disease in children

**DOI:** 10.3389/fped.2023.1205971

**Published:** 2023-07-19

**Authors:** L. Wannaz, L. Boillat, M. H. Perez, S. Di Bernardo

**Affiliations:** ^1^ Pediatric Cardiology, Women-Mother-Child Department, Lausanne University Hospital and University of Lausanne, Lausanne, Switzerland; ^2^ Pediatric Intensive Care Unit, Women-Mother-Child Department, Lausanne University Hospital and University of Lausanne, Lausanne, Switzerland

**Keywords:** congenital heart disease, cardiac surgery, intensive care, low cardiac output syndome, levosimendan

## Abstract

**Introduction:**

Low cardiac output syndrome (LCOS) is a significant cause of morbidity and the leading cause of mortality after pediatric cardiac surgery. Levosimendan has been shown safe and effective in pediatrics to treat LCOS. We aimed to review our local strategy with preoperative prophylactic Levosimendan infusion to minimize LCOS after heart surgery in identified high-risk patients.

**Methods:**

Retrospective monocentric study. As there is no reliable cardiac output measurement in children, we recorded hemodynamic parameters as surrogates of cardiac output after extracorporeal circulation through an electronic patient survey system at different time points.

**Results:**

Seventy-two children received Levosimendan before surgery between 2010 and 2019. As expected, most patients were newborns and infants with prolonged open-heart surgeries. Median cardiopulmonary bypass time was 182 [137–234] min, and aortic clamping time was 95 [64–126] min. The postoperative hemodynamic parameters, vasoactive-inotropic score, and urine output remained stable throughout the first 48 h. Only a tiny portion of the patients had combined surrogate markers of LCOS with a maximal median arterial lactate of 2.6 [1.9–3.5] mmol/L during the first six postoperative hours, which then progressively normalized. The median arterio-venous difference in oxygen saturation was 31 [23–38] % between 12 and 18 h post-surgery and gradually decreased. The median venous-to-arterial CO2 difference was the highest at 10 [7–12] mmHg between 12 and 18 h post-surgery. Nine patients (13%) required extracorporeal membrane oxygenation. No patient required dialysis or hemofiltration. Mortality was 0%.

**Conclusion:**

Before congenital heart surgery, preoperative prophylactic administration of Levosimendan seems effective and safe for decreasing occurrence and duration of LCOS in high-risk children.

## Introduction

Low cardiac output syndrome (LCOS) defines a post-surgical state where the imbalance between oxygen supply and demand in the body results in hypoxemia, end-organ ischemia, and multiple organ failure. LCOS is an important cause of morbidity, leading to prolonged mechanical ventilation (MV) and hospital stay, and one of the leading causes of mortality after pediatric cardiac surgery (1–3). The literature evaluates its incidence as up to 25%, mainly between 6 and 18 h after surgery (3, 4). LCOS can be explained by cardioplegia, reperfusion-ischemia, inflammatory process, and pulmonary and systemic vascular resistance alteration due to extracorporeal circulation (ECC) (4–6). The underlying physiologic alteration from congenital heart disease also should not be forgotten in LCOS development. Major risk factors for developing LCOS are multiple and well-described (7, 8). The principal ones are related either to cardiac surgery (technical difficulty of the surgery; long cardiopulmonary bypass (CPB) time (≥60 min) and long aortic cross-clamping (ACC) time) or to the basal characteristics of the patient at surgery (young age (≤4 years old), hypoxemia (≤93% of oxygen saturation), myocardial dysfunction, severe right ventricular hypertrophy). Non-invasive techniques in pediatrics cannot precisely measure cardiac output (4, 9). Therefore, in children, LCOS diagnostic is based on a pool of hemodynamic parameters and clinical signs used as surrogates of cardiac output. In literature, the most frequently used parameters are tachycardia, hypotension, poor perfusion, oliguria, low central venous oxygen saturation (SvO2), high venous-to-arterial pCO2 difference (DavCO2), increased arterial lactate level, and an increase in vasoactive-inotropic support (3, 6, 10).

Levosimendan was first described in 1995 by Haikala et al. (11); its action stabilizes the cardiac troponin C conformation after binding with calcium, enhancing cardiomyocyte contraction (11–13). Levosimendan's inotrope activity results in a dose-dependent improvement of cardiac output, stroke volume, and heart rate. It demonstrates a vasodilatative action and reduces cytokine levels by activating K-ATP channels (14–17). Contrary to other calcium sensitizers, Levosimendan does not extend the cardiomyocyte relaxation phase and therefore does not impair diastolic function (18–20). The pharmacological profile of Levosimendan prevents the development of classical secondary effects of other vasoactive-inotropic drugs like an increase in oxygen consumption from the myocardium, tachyarrhythmia, tachyphylaxis, and desensitization of the adrenergic pathway (3, 6, 11, 12, 21). Levosimendan's pharmacokinetics is characterized by a short half-life primary molecule (1 h) transformed through the liver into an active metabolite (OR-1896) with a longer half-life (3 days). Then 24-hour perfusion of Levosimendan could generate effects lasting 1 to 2 weeks (22, 23). The pharmacological properties of Levosimendan have been demonstrated to be similar in children and adults, allowing its use in younger patients (24, 25). In 2017, a Cochrane study reviewed its prophylactic use in pediatric patients undergoing surgery for congenital heart disease. The authors conclude that the current level of evidence is insufficient to judge whether prophylactic Levosimendan prevents low cardiac output syndrome and mortality (26).

In this unclear context, we aimed to review our local strategy with preoperative prophylactic Levosimendan infusion in high-risk pediatric patients to minimize LCOS after heart surgery.

## Material and methods

Our study is retrospective and descriptive. It takes place in the pediatric intensive care unit (PICU) of Lausanne University Center, Switzerland. It is a tertiary care teaching center with approximately 200 pediatric open-heart surgeries annually.

### Study design and patients

We reviewed nine years (2010–2019) of prophylactic Levosimendan administration for cardiac surgery in our PICU. We selected all cardiac surgery patients who received Levosimendan before cardiac surgery, apart from interventions without CPB, heart transplantation or ventricular assist device implantation. In our centre, only patients at high-risk for LCOS receive prophylactic Levosimendan. The use of prophylactic Levosimendan is decided on objective factors identified as well as in literature than in our background, like CPB time ≥180 min, ACC time ≥90 min, systolic dysfunction before surgery and severe left or right ventricular hypertrophy with diastolic dysfunction, newborns and infants, but also relies on our interdisciplinary team expertise and discussion between cardiologists, intensivists, anesthesiologists and cardiac surgeon. When circulatory support with extracorporeal membrane oxygenation (ECMO) was required after surgery, its data were included in the population descriptions, but its LCOS data were not analyzed. Levosimendan's administration protocol was identical for every patient, with a total administration of 0.1 mcg/kg/min for 48 h. Initially, the infusion was started 24–48 h before surgery, stopped during CPB time, and pursued after CPB weaning until the total 48-hour infusion was completed. With gaining experience in this set-up, the Levosimendan's perfusion was initiated 12 h before open-heart surgery, thus avoiding hypotension at induction due to the synergic effect between Levosimendan and anesthetic drugs, and pursued the next 36 h after the operation. Our centre's standard vasoactive-inotropic and postoperative strategy was published in 2017 (27). After Levosimendan infusion, Milrinone is started as a continuous infusion with a dose of 0.5 mcg/kg/min and increased to maximal 0.75 mcg/kg/min for inotropic support if needed. The vasopressor support with Norepinephrine is adjusted for maintenance of perfusion pressure if needed, associated with low-dose Dopamine (3 mcg/kg/min). Electrolytes are strictly controlled, and MV or non-invasive positive pressure ventilation (NIPPV) is routinely used. Our center also has a qualified team to set up and manage ECMO if needed.

All surrogate parameters for LCOS were retrospectively collected through an electronic patient survey system (Metavision5®, iMDsoft, Israel). Since admission to PICU, this patient data management system (PDMS) collects vital signs in real-time, minute-to-minute, such as ventilation, hemodynamic parameters, and diuresis. It also records all laboratory findings as well as drug administration. We also reviewed the operative protocols and hospitalization letters through electronic medical records (Soarian®, Cerner) and echocardiographic data through our ultrasonography program (IntelliSpace Cardiovascular®). Vitals signs, LCOS parameters, laboratory findings, and drug administrations were analyzed at 6-hour intervals until five days postoperatively. T0 was considered to be the end of the CPB time.

We collected the following data from different sources for the LCOS evaluation: heart rate, mean arterial pressure, mean central venous pressure, hourly urine output, arterial blood lactate, arterial hemoglobin saturation, central venous hemoglobin saturation, arterial CO2 and central venous CO2. The maximal rate of vasoactive drugs was collected for the calculation of the Vasoactive-Inotropic Score (VIS), score broadly used and already described elsewhere (28). To assess LCOS incidence in our population, we created a score based on established LCOS criteria of Hummel's Cochrane review (26) and PRIMACORP trial (3).

Our local ethics committee approved this study and waived the informed consent.

### Statistical analysis

The statistical analysis was performed with Stata 16.0 (StatCorp®). The continuous parameters are presented as median [interquartile range], and discrete parameters as absolute numbers (percentage). Comparison of continuous data was analyzed with the Wilcoxon-Mann-Whitney test and by more than two with the Dunn's test. Multiple repeated measurements were analyzed using the ANOVA test with Bonferroni post-test inference. Discrete variables were analyzed using Fisher's exact test. Statistical significance was inferred at a value of *p* < 0.05.

## Results

### Population description

Demographic data are shown in Table 1. 72 patients were included for the analysis with an equal representation of males (47%) and females (53%). The majority were newborns and infants, with a median age of 0.5 [0.2–9.2] months and a median weight of 3.5 [2.9–7.9] Kg. RACHS scores were high (68% of the patients with a RACHS ≥ 4), with the principal diagnosis of transposition of the great arteries (29%), single ventricle (18%), and right ventricular outflow tract obstruction (18%). Perioperative patient's data are shown in Table 2. The median CPB time was 182 [137–234] min, with a median ACC time of 95 [64–126] min. Circulatory arrest with preserved cerebral perfusion was necessary for 17 patients (24%), and the median minimal body temperature of 32 [25–34] °C was measured during CPB. Five patients (7%) were weaned from MV immediately after surgery in the operating room. The remaining patients had a median MV time of 7.7 [3.0–12.7] days. In 49 patients (68%), MV was relayed by NIPPV for a median time of 23.2 [17.0–35.2] hours. The median length of stay in pediatric intensive care was 16.7 [10–41] days.

**Table 1:** Demographic data.

	*N* = 72
Sex	
Male	34 (47)
Female	38 (53)
Age (months)	0.5 [0.2–9.2]
Weight (kg)	3.5 [2.9–7.9]
Height (cm)	51 [50–66]
BSA (m2)	0.21 [0.20–0.34]
Cardiac anomaly	
TGA	20 (29)
RVOTO	13 (18)
SV	13 (18)
Aortic arch pathology	10 (14)
Other	15 (21)
RACHS score	
RACHS 2	7 (11)
RACHS 3	14 (21)
RACHS 4	35 (52)
RACHS 5 + 6	11 (16)

Data are expressed as *N* (%) or P50 [P25-P75].

BSA, body surface area; TGA, transposition of the great arteries; RVOTO, right ventricular outflow tract obstruction; SV, single ventricle; RACHS, risk-adjusted classification for congenital heart surgery.

**Table 2:** Perioperative data.

	*N *= 72
CPB time (minutes)	182 [137–234]
ACC time (minutes)	95 [64–126]
ECMO	9 (12)
(Days)	5 [4–6]
Cooling system	16 (22)
(Hours)	5 [2–19]
Open chest	40 (56)
(Days)	4 [3–6]
MV	67 (93)
(Days)	8 [3–13]
NIPPV	49 (68)
(Hours)	23 [17–35]
In-hospital stay (days)	17 [10–41]
Preoperative EF (%)	65 [60–68]
Postoperative EF (%)	52 [45–62]
5-days postoperative EF (%)	60 [55–65]
Preoperative SF (%)	39 [30–47]
Postoperative SF (%)	25 [20–32]
5-days postoperative SF (%)	32 [27–37]

Data are expressed as *N* (%) or P50 [P25-P75].

CPB, cardiopulmonary bypass; ACC, aortic cross-clamping; ECMO, extracorporeal membrane oxygenation; MV, mechanical ventilation; NIPPV, non-invasive positive pressure ventilation; EF, ejection fraction; SF, shortening fraction.

### Complications

Weaning of CPB was not possible in nine patients (13%), and they required ECMO with a median support time of 5.4 [4.4–6.0] days. In thirty-two patients (51%), delayed chest closure occurred after a median time of 4.2 [2.9–6.2] days. No patient required dialysis or hemofiltration. Sixteen patients (22%) required the use of a cooling system due to postoperative hyperthermia or temperature control for a median of 5 [1.7–18.6] hours. Mortality was 0%.

### Outcome analysis

**Figure 1:**
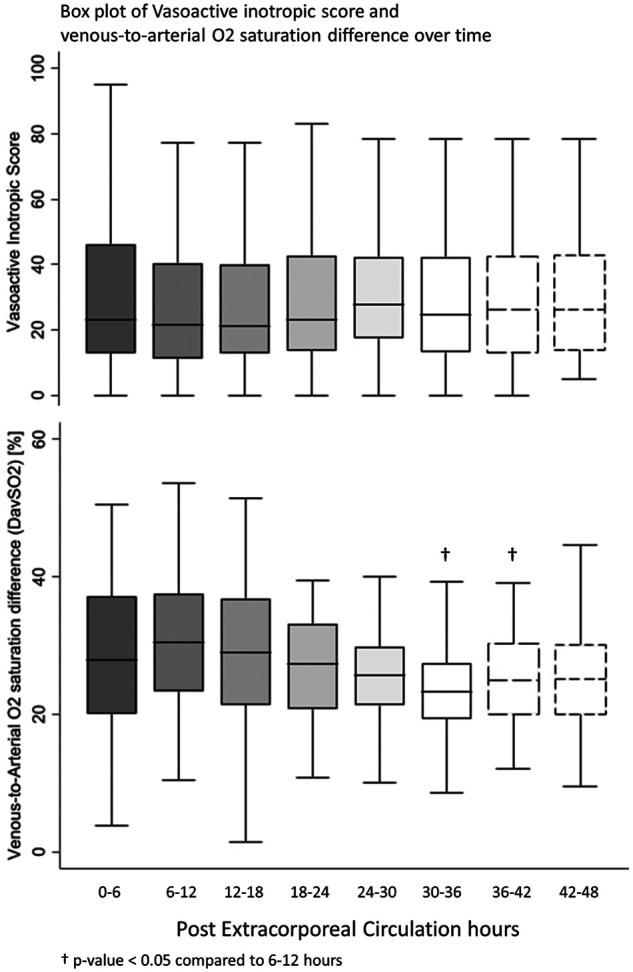
Box plot of vasoactive inotropic score and venous-to-arterial O2 saturation difference over time.

**Figure 2:**
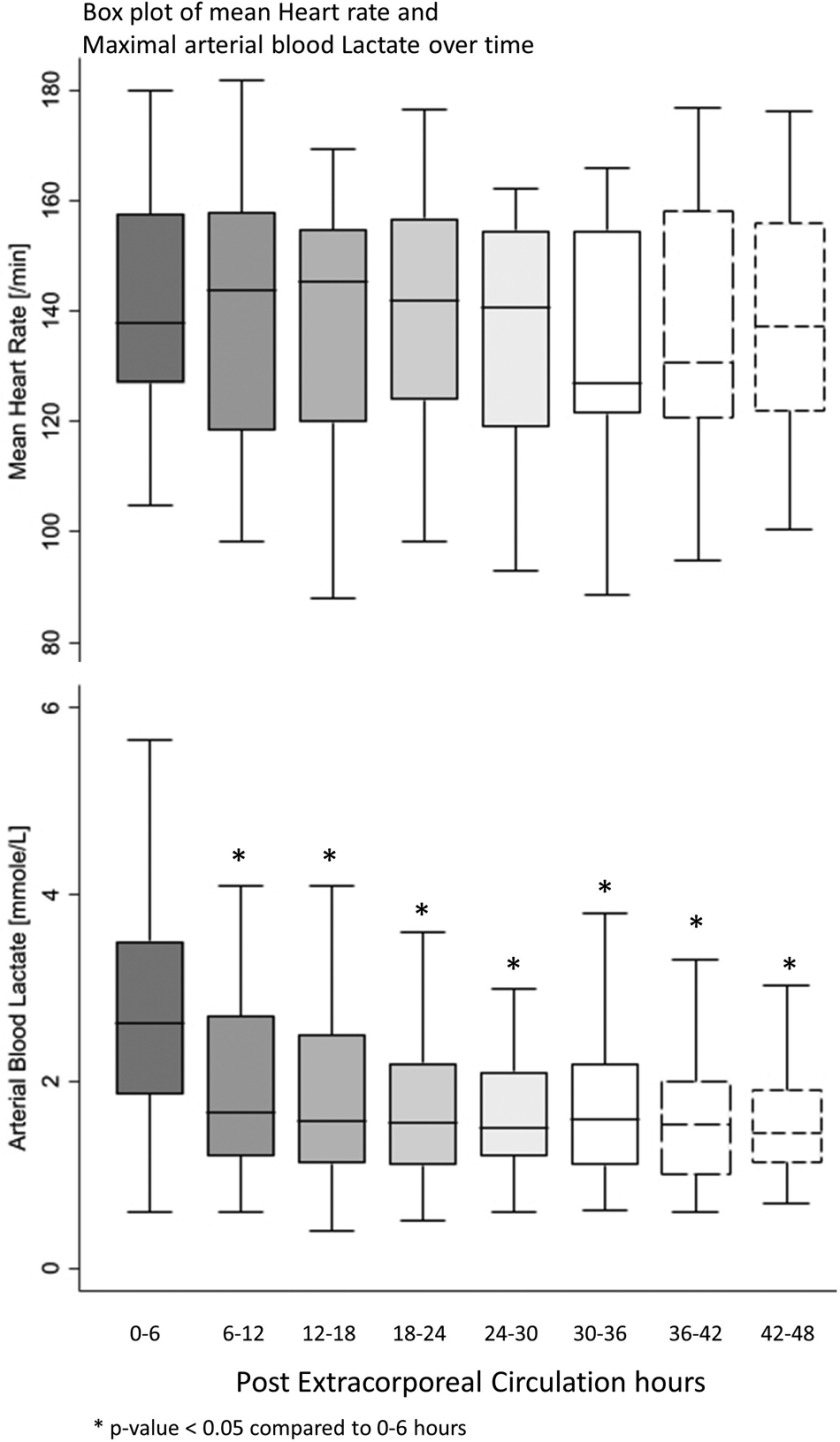
Box plot of mean heart rate and maximal arterial blood lactate over time.

**Figure 3:**
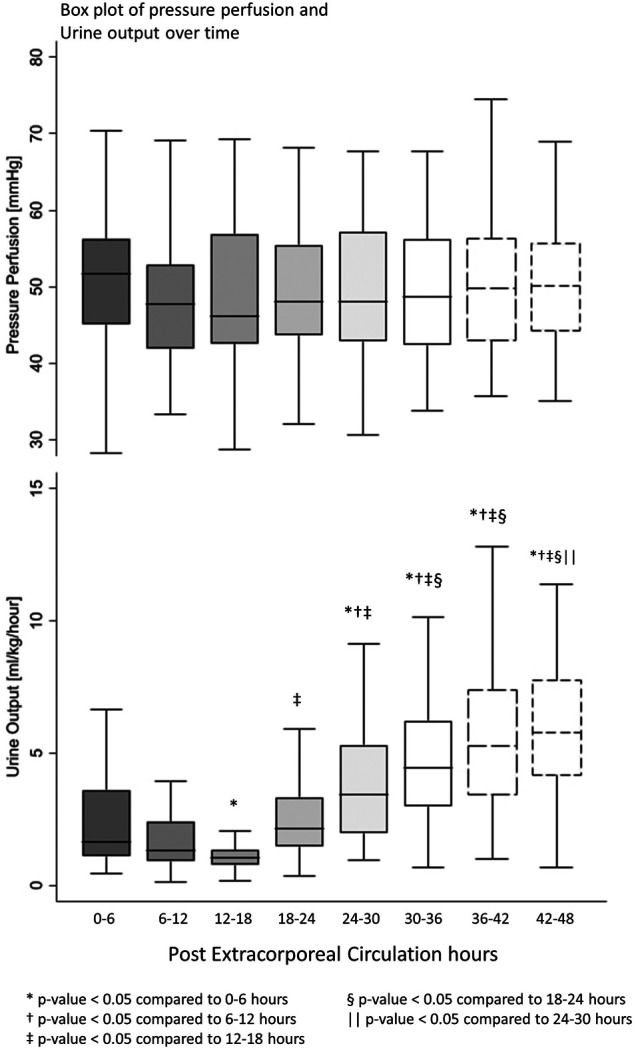
Box plot of pressure perfusion and urine output over time.

All postoperative patients had inotrope support with Levosimendan followed by Milrinone and vasoactive support with Norepinephrine, and Dopamine. LCOS parameters analysis are shown in Table 3. No escalation of aminergic support was required after surgery, represented by stability in VIS during the first 48 h postoperative with a median between 21 [13–40] in the first 6 h postoperative and 28 [17–42] (not statistically significant) (Figure 1). Norepinephrine being our commonly used amine, its median of maximal level during the first 6 h postoperative per patient was 0.24 [0.02–0.45] mcg/kg/min, then decreased to 0.20 [0.11–0.41] at 48 h. Median venous-to-arterial O2 saturation difference (DavSO2) was maximal between 12 and 18 h after surgery with 31 [23–38] % [median SvO2 value of 63 (58–70) %] and decreased gradually after that (Figure 1). Arterial lactate was maximal in the first 6 h after surgery with a median of 2.6 [1.9–3.5] mmol/L, then decreased significantly to normal values <2 mmol/L (Figure 2). Urine output was preserved after surgery (>1 ml/kg/h) without a significant drop and significantly increased after 12–18 h (Figure 3). DavCO2 was the highest at 10 [7–12] mmHg between 12 and 18 h post-surgery, then showed a significant decrease with stabilization at 36–42 h after surgery. Postoperative median heart rate (Figure 2), median mean arterial pressure, median central venous pressure, and median perfusion pressure (Figure 3) were stable without any significant change, with respective postoperative values between 12 and 18 h of 145 [120–155] bpm, 55 [48–65] mmHg, 8 [7–10] mmHg, and 46 [42–57] mmHg. Only a tiny portion (5%–8%) of the patients demonstrated combined surrogate markers of LCOS at intervals.

**Table 3:** Postoperative LCOS parameters (hours post end of extracorporeal circulation).

	0–6 h	6–12 h	12–18 h	18–24 h	24–30 h	30–36 h	36–42 h	42–48 h	*p-value*
Mean HR (bpm)	137 [127–158]	144 [118–158]	145 [120–155]	142 [124–157]	141 [119–155]	127 [121–155]	131 [121–158]	137 [122–156]	*0*.*9640*
Mean BP (mmHg)	59 [55–65]	57 [49–66]	55 [48–65]	57 [52–65]	57 [50–66]	57 [49–65]	57 [51–65]	57 [52–65]	*0*.*7436*
CVP (mmHg)	8 [6–10]	8 [6–10]	8 [7–10]	8 [7–11]	8 [6–10]	8 [7–10]	7 [6–10]	8 [6–11]	*0*.*8228*
PP (mmHg)	52 [45–56]	48 [42–53]	46 [42–57]	48 [44–55]	48 [43–57]	49 [42–56]	50 [43–56]	50 [44–56]	*0*.*7204*
VIS	23 [13–46]	22 [11–40]	21 [13–40]	23 [14–43]	28 [17–42]	25 [13–42]	26 [13–43]	26 [14–43]	*0*.*8412*
Urine output (ml/kg/hour)	1.6 [1.1–3.6]	1.3 [0.9–2.4]	1.0 [0.8–1.3]*	2.2 [1.5–3.3]^‡^	3.4 [2.0–5.3]*^†‡^	4.4 [3.0–6.2]*^†‡§^	5.3 [3.4–7.4]*^†‡§^	5.8 [4.2–7.8]*^†‡§||^	***0***.***0001***
Maximal arterial lactate (mmol/L)	2.6 [1.9–3.5]	1.7 [1.2–2.7]*	1.6 [1.1–2.5]*	1.6 [1.1–2.2]*	1.5 [1.2–2.1]*	1.6 [1.1–2.2]*^‡^	1.5 [1.0–2.0]*	1.5 [1.1–1.9]*	***0***.***0001***
DavSO2 (%)	28 [20–37]	31 [23–38]	29 [21–37]	27 [21–33]	26 [21–30]	23 [19–27]^†^	25 [20–30]^†^	25 [20–30]	***0***.***0049***
DavCO2 (mmHg)	8 [3–12]	10 [7–12]	10 [7–12]	8 [5–10]	8 [6–10]	8 [6–10]	8 [5–10]^†^	7 [5–9]^†^	***0***.***0047***

Data are expressed as P50 [P25-P75]. LCOS, low cardiac output syndrome; HR, heart rate; BP, blood pressure; CVP, central venous pressure; PP, perfusion pressure; VIS, vasoactive-inotropic score; DavSO2, venous-to-arterial O2 saturation difference; DavCO2, venous-to-arterial pCO2 difference.

Significant *p-value for paired comparison with Dunn's test*:

* Compared to 0–6 h.

^†^ Compared to 6–12 h.

^‡^ Compared to 12–18 h.

^§^ Compared to 18–24 h.

^||^ Compared to 24–30 h.

Significant value of *p* < 0.05 in bold.

## Discussion

Our data show that prophylactic Levosimendan seems effective in controlling cardiac output as postoperative hemodynamic parameters remained stable and improved throughout the first postoperative 48 h, as well as the vasoactive-inotropic score and the urine output. Only a small portion of the patients demonstrated combined surrogate markers of LCOS during these 48 first hours. In our study, Levosimendan's infusion reduces the escalation of catecholamines, hence avoiding their secondary effects such as increased myocardial oxygen consumption, tachycardia, arrhythmia, tachyphylaxis, and desensitization (29). Despite the stability of the VIS, we note that its value is relatively high in our center, as our prime amine is Norepinephrine, which has a high weighting in VIS. Nevertheless, neither dialysis nor hemofiltration was required in our population. Volume expansion with crystalloid infusion is relatively low and decreases right after surgery, supporting that even after a long CPB time, cardiac output is adequate and inflammatory vasoplegia controlled. Previous studies on pediatric prophylactic Levosimendan in cardiac surgery support our results of stability in clinical or laboratory hemodynamic state and prevention of LCOS. However, most showed no evidence on other endpoints such as MV time, PICU length of stay, or mortality. Osthaus et al. (30) showed lactate and SvO2 improvement in children receiving intraoperative levosimendan but did not analyze an LCOS or mortality endpoint. Egan et al. (31) report an improvement in arterial lactate, heart rate, mean arterial pressure, and the reduction of aminergic support in a pediatric group with established LCOS and confirm higher benefits in the second group, receiving prophylactic Levosimendan before the end of CPB. In 2020, a systematic review and meta-analysis with seven pediatric studies (32) concluded in the reduction in LCOS incidence after Levosimendan infusion compared to other inotrope or placebo; however, without significant improvement on other outcomes such as the duration of MV, PICU length of stay, or mortality. Wang et al. (33) showed a clear improvement in cardiac cycle efficiency (a novel indicator of hemodynamic performance) with prophylactic Levosimendan in children, but without effect on LCOS or mortality. Hummel's Cochrane review (26) includes five pediatric studies and reconstructs the number of patients experiencing LCOS according to their score: no effect on LCOS incidence, MV duration, PICU length of stay, or mortality was found. However, the patient population is inhomogenous in all these different studies (including simple and complex cardiac surgery), and the potentially favorable effect of prophylactic Levosimendan is then underestimated; according to the local experience, our policy is to anticipate which patient is at higher risk of developing post-surgical low cardiac output.

Based on our experience with Levosimendan in rescue following open-heart surgery in children (34) and the disappointing literature results, only high-risk patients for postoperative LCOS enter the prophylactic Levosimendan protocol. This selection is made preoperatively by a multidisciplinary team including pediatric cardiologists, intensivists, cardiac surgeons, and anesthetists and is based on known LCOS risk factors in our center. This selection explains the overrepresentation of newborns and infants, the high RACHS scores in most of our patients, the long CPB and ACC time, and the lower standard limit for preoperative ejection shortening fraction. Regarding their own experience and expertise, other teams should evaluate which patient is at high risk of LCOS and should benefit from prophylactic Levosimendan. Despite some contradicting results (35), some data demonstrate the beneficial effect of prophylactic Levosimendan in adults, and it seems more relevant for patients at high risk for postoperative LCOS and those with previous myocardial dysfunction (36–40). Based on these previous results and on the study of Ricci et al., which, showed benefit of prophylactic Levosimendan in a population of newborns with high RACHS scores (41), a prospective multicenter randomized controlled pediatric study selecting patients at high risk for postoperative LCOS could potentially demonstrate the beneficial effect of prophylactic Levosimendan.

After cardiac surgery, mortality rate is globally around 3.2% but around 7% in neonates related to the complexity of the diagnosis and procedure, the patient's weight, and the presence of a univentricular status (42). It is important to emphasize that according to important registry papers, the mortality rate decreased clearly over the last thirty years and is related to the volume of pediatric cardiac surgery performed in a center (43). Even if most of the teams did not show mortality reduction in patients receiving prophylactic Levosimendan, in our study mortality is very low, despite a large majority of newborns and infants with complex diagnoses and procedures and in a relatively small volume center.

The best timing for the onset of Levosimendan's infusion is uncertain, although two other teams used Levosimendan in the preoperative course in pediatrics (44, 45). However, to our knowledge, our study is the largest, using a conditioning protocol starting 24–48 h before surgery, with most patients having the infusion started about 12 h before CPB. Most other teams in pediatrics use prophylactic Levosimendan perioperative or immediately after surgery, as described in Cochrane 2017 (26). Tasouli et al. (46) showed the superiority in adult patients for an early onset of Levosimendan infusion in the operating room versus a later onset in the intensive care unit (ICU). Levosimendan improved hemodynamic parameters in both groups, but the ICU and hospital stay lengths were lower in the early-onset group. Another paper from Eriksson et al. reports that an intraoperative Levosimendan infusion facilitates weaning from CPB in adults (47). Considering this statement and the pharmacokinetics of Levosimendan, we choose our protocol to start before surgery. Our strategy may also allow intraoperative cardiac improvement in high-risk patients, possibly leading to a better postoperative outcome. Another fact favoring a preoperative administration of Levosimendan is that its plasma concentration may be lower in postoperative patients (48). This variability may probably be explained as Levosimendan pharmacokinetic varies in the postoperative course in several presumed ways. For example, diuretics, which are almost systematically used in the postoperative cardiac course, may increase urine excretion; antibiotics and postoperative immobilization may affect the gastrointestinal system, which reduces Levosimendan's metabolite and hypoalbuminemia, often noticed in the postoperative course of heart surgery, may decrease its transportation in plasma.

### Study’s limitation

Our study has several limitations, including the retrospective setting and a single-center experience. The retrospective aspect should be offset because our electronic patient survey system collects real-time, minute-to-minute information, allowing precise analyses. Patients requiring immediate circulatory support with ECMO were excluded from LCOS analyses because the ECMO support skews all the parameters analyzed for determining the LCOS. ECMO support—for hemodynamic or respiratory reasons—can be defined as a severe LCOS after a bypass or consecutive pulmonary-myocardial severe dysfunction. Nonetheless, for this selected group of patients, ECMO support is somewhat not infrequent but all patients were weaned from ECMO support and survived.

As frequently encountered in congenital heart disease studies, and though the study group is selected, there is heterogeneity in patients regarding cardiac anatomy, type of surgery, and age. Obviously, more than one cardiac surgeon performed surgeries, and different techniques may have been used. Concerning surgical procedures, we also should emphasize that our study is based on nine years of experience, during which technical and material aspects of cardiac surgery have possibly improved or changed. The prophylactic administration of Levosimendan is based in our centre on reproducible parameters as long-lasting ECC or ACC, or other patients characteristics like systolic dysfunction or severe ventricular hypertrophy. Other aspects of deciding to initiate prophylactic Levosimendan are subjective and depend on the local team experience or the types of patients recruited in the centre. Each centre should address the special condition of patients referred for surgery and decide which would benefit from Levosimendan preoperatively. This experience cannot be reproduced from our. Although the sample size could be considered small, it is, to our knowledge, one of the largest cohorts studying preoperative prophylactic Levosimendan in children. Only a few single centers have the caseload of patients with congenital heart disease to conduct a powerful study.

### Conclusion and future

Based on our analysis and observation, prophylactic administration of Levosimendan preoperatively seems safe and promising. As no control group was used in our study, we cannot prove that our strategy is effective in controlling LCOS in selected patients, but many clues point in this direction: parameters used to evaluate LCOS in a clinical context are stable in the first postoperative days, needing no additional hemodynamic support unless the patient is on ECMO support after the CPB. In our opinion, this strategy for high-risk patients needing elective complex heart surgery can be used safely, but each centre/team should determine the high-risk patients in its centre. Further studies would be warranted to define more precisely the children who would benefit from Levosimendan preoperatively and the advantage over placebo or other strategies.

## Data Availability

The raw data supporting the conclusions of this article will be made available by the authors, without undue reservation.

## References

[1] Ma M Gauvreau K Allan CK Mayer JE Jenkins KJ. Causes of death after congenital heart surgery. Ann Thorac Surg. (2007) 83(4):1438–45. 10.1016/j.athoracsur.2006.10.073 17383354

[2] Shi S Zhao Z Liu X Shu Q Tan L Lin R Perioperative risk factors for prolonged mechanical ventilation following cardiac surgery in neonates and young infants. Chest. (2008) 134(4):768–74. 10.1378/chest.07-2573 18625673

[3] Hoffman TM Wernovsky G Atz AM Kulik TJ Nelson DP Chang AC Efficacy and safety of milrinone in preventing low cardiac output syndrome in infants and children after corrective surgery for congenital heart disease. Circulation. (2003) 107(7):996–1002. 10.1161/01.CIR.0000051365.81920.28 12600913

[4] Wernovsky G Wypij D Jonas RA Mayer JE Hanley FL Hickey PR Postoperative course and hemodynamic profile after the arterial switch operation in neonates and infants. A comparison of low-flow cardiopulmonary bypass and circulatory arrest. Circulation. (1995) 92(8):2226–35. 10.1161/01.CIR.92.8.2226 7554206

[5] Bailey JM Hoffman TM Wessel DL Nelson DP Atz AM Chang AC A population pharmacokinetic analysis of milrinone in pediatric patients after cardiac surgery. J Pharmacokinet Pharmacodyn. (2004) 31(1):43–59. 10.1023/B:JOPA.0000029488.45177.48 15346851

[6] Chandler HK Kirsch R. Management of the low cardiac output syndrome following surgery for congenital heart disease. Curr Cardiol Rev. (2016) 12(2):107–11. 10.2174/1573403X12666151119164647 26585039 PMC4861938

[7] Jenkins KJ Gauvreau K Newburger JW Spray TL Moller JH Iezzoni LI. Consensus-based method for risk adjustment for surgery for congenital heart disease. J Thorac Cardiovasc Surg. (2002) 123(1):110–8. 10.1067/mtc.2002.119064 11782764

[8] Song B Dang H Dong R. Analysis of risk factors of low cardiac output syndrome after congenital heart disease operation: what can we do. J Cardiothorac Surg. (2021) 16(1):135. 10.1186/s13019-021-01518-7 34001213 PMC8130417

[9] Parr GV Blackstone EH Kirklin JW. Cardiac performance and mortality early after intracardiac surgery in infants and young children. Circulation. (1975) 51(5):867–74. 10.1161/01.CIR.51.5.867 235375

[10] Jothinath K Balakrishnan S Raju V Menon S Osborn J. Clinical efficacy of levosimendan vs milrinone in preventing low cardiac output syndrome following pediatric cardiac surgery. Ann Card Anaesth. (2021) 24(2):217–23. 10.4103/aca.ACA_160_19 33884979 PMC8253017

[11] Haikala H Levijoki J Linden IB. Troponin C-mediated calcium sensitization by levosimendan accelerates the proportional development of isometric tension. J Mol Cell Cardiol. (1995) 27(10):2155–65. 10.1016/S0022-2828(95)91371-8 8576932

[12] Endoh M. Mechanisms of action of novel cardiotonic agents. J Cardiovasc Pharmacol. (2002) 40(3):323–38. 10.1097/00005344-200209000-00001 12198318

[13] Sorsa T Heikkinen S Abbott MB Abusamhadneh E Laakso T Tilgmann C Binding of levosimendan, a calcium sensitizer, to cardiac troponin C. J Biol Chem. (2001) 276(12):9337–43. 10.1074/jbc.M007484200 11113122

[14] Pataricza J Hohn J Petri A Balogh A Papp JG. Comparison of the vasorelaxing effect of cromakalim and the new inodilator, levosimendan, in human isolated portal vein. J Pharm Pharmacol. (2000) 52(2):213–7. 10.1211/0022357001773715 10714952

[15] Yokoshiki H Katsube Y Sunagawa M Sperelakis N. Levosimendan, a novel Ca2+ sensitizer, activates the glibenclamide-sensitive K+ channel in rat arterial myocytes. Eur J Pharmacol. (1997) 333(2–3):249–59. 10.1016/S0014-2999(97)01108-4 9314042

[16] Papp Z Csapo K Pollesello P Haikala H Edes I. Pharmacological mechanisms contributing to the clinical efficacy of levosimendan. Cardiovasc Drug Rev. (2005) 23(1):71–98. 10.1111/j.1527-3466.2005.tb00158.x 15867949

[17] Parissis JT Adamopoulos S Antoniades C Kostakis G Rigas A Kyrzopoulos S Effects of levosimendan on circulating pro-inflammatory cytokines and soluble apoptosis mediators in patients with decompensated advanced heart failure. Am J Cardiol. (2004) 93(10):1309–12. 10.1016/j.amjcard.2004.01.073 15135713

[18] Givertz MM Andreou C Conrad CH Colucci WS. Direct myocardial effects of levosimendan in humans with left ventricular dysfunction: alteration of force-frequency and relaxation-frequency relationships. Circulation. (2007) 115(10):1218–24. 10.1161/CIRCULATIONAHA.106.668640 17339544

[19] Michaels AD McKeown B Kostal M Vakharia KT Jordan MV Gerber IL Effects of intravenous levosimendan on human coronary vasomotor regulation, left ventricular wall stress, and myocardial oxygen uptake. Circulation. (2005) 111(12):1504–9. 10.1161/01.CIR.0000159252.82444.22 15781741

[20] Kaheinen P Pollesello P Levijoki J Haikala H. Effects of levosimendan and milrinone on oxygen consumption in isolated Guinea-pig heart. J Cardiovasc Pharmacol. (2004) 43(4):555–61. 10.1097/00005344-200404000-00011 15085067

[21] Silvetti S Silvani P Azzolini ML Dossi R Landoni G Zangrillo A. A systematic review on levosimendan in paediatric patients. Curr Vasc Pharmacol. (2015) 13(1):128–33. 10.2174/1570161112666141127163536 25440597

[22] Kivikko M Antila S Eha J Lehtonen L Pentikainen PJ. Pharmacodynamics and safety of a new calcium sensitizer, levosimendan, and its metabolites during an extended infusion in patients with severe heart failure. J Clin Pharmacol. (2002) 42(1):43–51. 10.1177/0091270002042001005 11808823

[23] Kivikko M Lehtonen L. Levosimendan: a new inodilatory drug for the treatment of decompensated heart failure. Curr Pharm Des. (2005) 11(4):435–55. 10.2174/1381612053382043 15725064

[24] Turanlahti M Boldt T Palkama T Antila S Lehtonen L Pesonen E. Pharmacokinetics of levosimendan in pediatric patients evaluated for cardiac surgery. Pediatr Crit Care Med. (2004) 5(5):457–62. 10.1097/01.PCC.0000137355.01277.9C 15329162

[25] Luther YC Schulze-Neick I Stiller B Ewert P Redlin M Nasseri B [Levosimendan-long-term inodilation in an infant with myocardial infarction]. Z Kardiol. (2004) 93(3):234–9. 10.1007/s00392-004-0053-9 15024592

[26] Hummel J Rucker G Stiller B. Prophylactic levosimendan for the prevention of low cardiac output syndrome and mortality in paediatric patients undergoing surgery for congenital heart disease. Cochrane Database Syst Rev. (2017) 8:CD011312. 10.1002/14651858.CD011312.pub3 28770972 PMC6483297

[27] Hosseinpour AR van Steenberghe M Bernath MA Di Bernardo S Perez MH Longchamp D Improvement in perioperative care in pediatric cardiac surgery by shifting the primary focus of treatment from cardiac output to perfusion pressure: are beta stimulants still needed? Congenit Heart Dis. (2017) 12(5):570–7. 10.1111/chd.12485 28580658

[28] Gaies MG Gurney JG Yen AH Napoli ML Gajarski RJ Ohye RG Vasoactive-inotropic score as a predictor of morbidity and mortality in infants after cardiopulmonary bypass. Pediatr Crit Care Med. (2010) 11(2):234–8. 10.1097/PCC.0b013e3181b806fc 19794327

[29] Namachivayam P Crossland DS Butt WW Shekerdemian LS. Early experience with Levosimendan in children with ventricular dysfunction. Pediatr Crit Care Med. (2006) 7(5):445–8. 10.1097/01.PCC.0000235251.14491.75 16885788

[30] Osthaus WA Boethig D Winterhalter M Huber D Goerler H Sasse M First experiences with intraoperative Levosimendan in pediatric cardiac surgery. Eur J Pediatr. (2009) 168(6):735–40. 10.1007/s00431-008-0834-7 18813947

[31] Egan JR Clarke AJ Williams S Cole AD Ayer J Jacobe S Levosimendan for low cardiac output: a pediatric experience. J Intensive Care Med. (2006) 21(3):183–7. 10.1177/0885066606287039 16672640

[32] Wang H Luo Q Li Y Zhang L Wu X Yan F. Effect of prophylactic levosimendan on all-cause mortality in pediatric patients undergoing cardiac surgery-an updated systematic review and meta-analysis. Front Pediatr. (2020) 8:456. 10.3389/fped.2020.00456 32923414 PMC7456871

[33] Wang A Cui C Fan Y Zi J Zhang J Wang G Prophylactic use of levosimendan in pediatric patients undergoing cardiac surgery: a prospective randomized controlled trial. Crit Care. (2019) 23(1):428. 10.1186/s13054-019-2704-2 31888711 PMC6937718

[34] Amiet V Perez MH Longchamp D Boulos Ksontini T Natterer J Plaza Wuthrich S Use of levosimendan in postoperative setting after surgical repair of congenital heart disease in children. Pediatr Cardiol. (2018) 39(1):19–25. 10.1007/s00246-017-1718-2 28884218

[35] Zhu J Zhang Y Chen L He Y Qing X. Levosimendan in patients with low cardiac output syndrome undergoing cardiac surgery: a systematic review and meta-analysis. Anaesth Crit Care Pain Med. (2019) 38(3):243–9. 10.1016/j.accpm.2018.08.005 30342103

[36] Harrison RW Hasselblad V Mehta RH Levin R Harrington RA Alexander JH. Effect of levosimendan on survival and adverse events after cardiac surgery: a meta-analysis. J Cardiothorac Vasc Anesth. (2013) 27(6):1224–32. 10.1053/j.jvca.2013.03.027 24050857

[37] Weber C Esser M Eghbalzadeh K Sabashnikov A Djordjevic I Maier J Levosimendan reduces mortality and low cardiac output syndrome in cardiac surgery. Thorac Cardiovasc Surg. (2020) 68(5):401–9. 10.1055/s-0039-3400496 31770777

[38] Lim JY Deo SV Rababa’h A Altarabsheh SE Cho YH Hang D Levosimendan reduces mortality in adults with left ventricular dysfunction undergoing cardiac surgery: a systematic review and meta-analysis. J Card Surg. (2015) 30(7):547–54. 10.1111/jocs.12562 25989324

[39] Sanfilippo F Knight JB Scolletta S Santonocito C Pastore F Lorini FL Levosimendan for patients with severely reduced left ventricular systolic function and/or low cardiac output syndrome undergoing cardiac surgery: a systematic review and meta-analysis. Crit Care. (2017) 21(1):252. 10.1186/s13054-017-1849-0 29047417 PMC5648477

[40] Tena MA Urso S Gonzalez JM Santana L Sadaba R Juarez P Levosimendan versus placebo in cardiac surgery: a systematic review and meta-analysis. Interact Cardiovasc Thorac Surg. (2018) 27(5):677–85. 10.1093/icvts/ivy133 29718383

[41] Ricci Z Garisto C Favia I Vitale V Di Chiara L Cogo PE. Levosimendan infusion in newborns after corrective surgery for congenital heart disease: randomized controlled trial. Intensive Care Med. (2012) 38(7):1198–204. 10.1007/s00134-012-2564-6 22527079

[42] Crowe S Brown KL Pagel C Muthialu N Cunningham D Gibbs J Development of a diagnosis- and procedure-based risk model for 30-day outcome after pediatric cardiac surgery. J Thorac Cardiovasc Surg. (2013) 145(5):1270–8. 10.1016/j.jtcvs.2012.06.023 22818122

[43] Allen SW Gauvreau K Bloom BT Jenkins KJ. Evidence-based referral results in significantly reduced mortality after congenital heart surgery. Pediatrics. (2003) 112(1 Pt 1):24–8. 10.1542/peds.112.1.24 12837863

[44] Abril-Molina A Gomez-Luque JM Perin F Esteban-Molina M Ferreiro-Marzal A Fernandez-Guerrero C Effect of preoperative infusion of levosimendan on biomarkers of myocardial injury and haemodynamics after paediatric cardiac surgery: a randomised controlled trial. Drugs R D. (2021) 21(1):79–89. 10.1007/s40268-020-00332-1 33367965 PMC7937581

[45] Pellicer A Riera J Lopez-Ortego P Bravo MC Madero R Perez-Rodriguez J Phase 1 study of two inodilators in neonates undergoing cardiovascular surgery. Pediatr Res. (2013) 73(1):95–103. 10.1038/pr.2012.154 23138399

[46] Tasouli A Papadopoulos K Antoniou T Kriaras I Stavridis G Degiannis D Efficacy and safety of perioperative infusion of levosimendan in patients with compromised cardiac function undergoing open-heart surgery: importance of early use. Eur J Cardiothorac Surg. (2007) 32(4):629–33. 10.1016/j.ejcts.2007.07.010 17702589

[47] Eriksson HI Jalonen JR Heikkinen LO Kivikko M Laine M Leino KA Levosimendan facilitates weaning from cardiopulmonary bypass in patients undergoing coronary artery bypass grafting with impaired left ventricular function. Ann Thorac Surg. (2009) 87(2):448–54. 10.1016/j.athoracsur.2008.10.029 19161758

[48] Wang W Zhou X Liao X Liu B Yu H. The efficacy and safety of prophylactic use of levosimendan on patients undergoing coronary artery bypass graft: a systematic review and meta-analysis. J Anesth. (2019) 33(4):543–50. 10.1007/s00540-019-02643-3 31025104

